# G-Networks to Predict the Outcome of Sensing of Toxicity

**DOI:** 10.3390/s18103483

**Published:** 2018-10-16

**Authors:** Ingrid Grenet, Yonghua Yin, Jean-Paul Comet

**Affiliations:** 1 University Côte d’Azur, I3S laboratory, UMR CNRS 7271, CS 40121, 06903 Sophia Antipolis CEDEX, France; Jean-Paul.Comet@univ-cotedazur.fr; 2 Intelligent Systems and Networks Group, Department of Electrical and Electronic Engineering, Imperial College, London SW7 2AZ, UK; y.yin14@imperial.ac.uk

**Keywords:** G-networks, random neural network, chemical compounds, machine learning, toxicity

## Abstract

G-Networks and their simplified version known as the Random Neural Network have often been used to classify data. In this paper, we present a use of the Random Neural Network to the early detection of potential of toxicity chemical compounds through the prediction of their bioactivity from the compounds’ physico-chemical structure, and propose that it be automated using machine learning (ML) techniques. Specifically the Random Neural Network is shown to be an effective analytical tool to this effect, and the approach is illustrated and compared with several ML techniques.

## 1. Introduction

G-Networks [1] are a family of queueing networks with a convenient and computationally efficient product form mathematical solution. The computation of the state of a G-Network is obtained via a simple fixed-point iteration, and the existence and uniqueness of the solution to the key G-Network state equation is easily verified [2]. G-Networks incorporate useful primitives, such as the transfer of jobs between servers or the removal of batches of jobs from excessively busy servers, which were developed in several successive papers including [3,4,5,6].

They have a wealth of diverse applications as a tool to analyse and optimise the effects of dynamic load balancing in large scale networks and distributed computer systems [7]. They are also used to model Gene Regulatory Networks [8,9]. A recent application of G-Networks is to the modelling of systems which operate with intermittent sources of energy, known as Energy Packet Networks [10,11,12,13,14,15].

The simplest version of G-Networks, known as the Random Neural Network (RNN) [16], has a powerful property of approximating continuous and bounded real-valued functions [17]. This property serves as the foundation for RNN based learning algorithms [18] and Deep Learning [19,20].

The RNN has been used for modelling natural neuronal networks [21], and for protein alignment [22]. It has been used with its learning algorithm [18] in several image processing applications including learning colour textures [23], the accurate evaluation of tumours from brain MRI scans [24] and the compression of still and moving images [25,26,27]. It was recently introduced as a tool for predicting the toxicity of chemical compounds [28].

In the field of computer network performance, the RNN has been used to build distributed controllers for quality of service routing in packet networks [29,30,31] and in the design of Software Defined Network controllers for the Internet [32,33]. Real-time optimised task allocation algorithms in Cloud systems [34,35] have also been built and tested. Recent applications have addressed the use of the RNN to detect network attacks [36] and attacks on Internet of Things (IoT) gateways [37].

In this paper, we introduce the use of the RNN and other ML techniques to reduce the use of in vivo laboratory experiments in the evaluation of the bioactivity and potential toxicity of chemical compounds. Indeed, the hope is that the toxicity of chemical compounds may in the future be determined through physical-chemical-computational means and processes, avoiding the use of laboratory animals.

Prediction in this area is challenging [38,39] because of high biological variability, especially when toxicity is the result from a sequence of causal factors. Therefore, we suggest that long-term toxicity prediction could be obtained by the prediction of in vitro bioactivity using chemical structure [40], followed by the prediction of in vivo effects from in vitro bioactivity [41,42].

Here, we only develop the first part of this challenge based on the RNN and other ML techniques to elucidate the quantitative structure–activity relationship (QSAR) [43] which predicts a compound’s activity using its physico-chemical properties and structural descriptors.

In Section 2, we present the data that we use and we discuss the techniques and performance metrics. In Section 3, we present some initial results obtained on a subset of data. Section 4 discusses the main conclusions.

## 2. RNN Based Learning and Other Methods

Since we need publicly available and agreed upon data in order to train and test the ML methods, including the RNN, we call upon the data released by the US Environmental Protection Agency (EPA) in the ToxCast database (https://www.epa.gov/chemical-research/exploring-toxcast-data, October 2015 release) which contains bioactivity data obtained for around 10,000 of compounds tested in more than several hundreds in vitro assays [44], and the Toxicity Reference database (ToxRefDB) with results from several types of in vivo studies for several hundreds of chemicals [45]. These data sets do not fully cover each other so that not all compounds tested in ToxCast are present in ToxRefDB.

We consider a subset of these data including compounds for which both in vitro and in vivo results are available. The subset selection follows three steps. First, we look for the overlap of compounds present both in ToxCast and ToxRefDB and having results for in vivo studies performed in rats during two years. We obtain a matrix with 418 compounds and 821 assays, with a lot of missing values. Secondly, we look for a large complete sub-matrix and we obtain a matrix of 404 compounds and 60 in vitro assays. Finally, in order to be sure to get a minimum of active compounds in the datasets, i.e., compounds for which an AC50 (half maximal activity concentration) could be measured, we remove assays with less than 5% of them and obtain a final matrix of 404 compounds and 37 assays.

For each of the 37 assays, we build a QSAR classification model to predict the bioactivity of a compound. These models use structural descriptors computed from the compound’s structure described in Structured Data Files. Two types of descriptors are used: (i) 74 physico-chemical properties (e.g., molecular weight, logP, etc.), which are continuous variables calculated using the RDKit Open-Source software [46] and normalized into the interval [0; 1] and (ii) 4870 fingerprints which are binary vectors representing the presence or absence of a chemical sub-structure in a compound [47]. The different types of fingerprints were generated using the pybel package in Python [48] and the PaDEL sofware [49] and are the following: FP3, Estate, KlekotaRoth, MACCS and PubChem fingerprints. Fingerprints being present in less than 5% of compounds are removed, leading to a final set of 731 fingerprints. Therefore, the obtained dataset is composed of 805 structural descriptors for the 404 compounds.

The property that we wish to predict, is the activity in each in vitro assay in a binarised form. It is generally measured as a AC50 value which is the dose of compound required to obtain 50% of activity in the assay. For compounds that were inactive in the assays, meaning that no AC50 could have been determined, an AC50 value of 1,000,000 mM have been used. In the following, we consider that the binary version of the activity is 0 for AC50 of 1,000,000 (meaning inactivity of the compound) and 1 otherwise.

### 2.1. Learning Algorithms

We recall that The Random Neural Network (RNN) is a simple version of the mathematical models called G-Networks, and that it represents the spiking (impulse-like) probabilistic behaviour of biological neural systems [50] which is a universal approximator for continuous and bounded functions [17]. It has a compact computationally efficient “product form solution”, so that, in steady-state, the joint probability distribution of the states of the neurons in the network can be expressed as the product of the marginal probabilities for each neuron. The probability that any cell is excited satisfies a nonlinear continuous function of the states of the other cells, and it depends on the firing rates of the other cells and the synaptic weights between cells. The RNN has been applied to many pattern analysis and classification tasks [26]. Gradient descent learning is often used for the RNN, but in this work we determine weights of the RNN using the cross-validation approach in [51].

The Multi Layer RNN (MLRNN) uses the original simpler structure of the RNN and investigates the power of single cells for deep learning [20]. It achieves comparable or better classification at much lower computation cost than conventional deep learning methods in some applications. A cross-validation approach is used to determine the structure and the weights and 20 trials are conducted to average the results. The structure of the MLRNN used here is fixed as having 20 inputs and 100 intermediate nodes.

Boosted Trees (called XGBoost in the sequel) is a popular tree ensemble method. The open-source software library XGBoost [52] provides an easy-to-use tool for implementing boosted trees with gradient boosting [53] and regression trees.

For the RNN and MLRNN we use the algorithms and software developed at Imperial College. For the XGBoost, we use the implementation and software explicitly mentioned in the references.

### 2.2. Classification Settings and Performance Metrics

For each of the 37 assays, we randomly subdivide the corresponding dataset *D* into a training set $D_{T}$ and a testing set $D_{t}$. From *D*, we randomly create 50 instances of $D_{T}$ and its complementary test set $D_{t}$ so that, for each instance, $D=D_{T}\cup D_{t}$. Each of the ML techniques listed above are first trained on each $D_{T}$ and then tested on $D_{t}$. The results we present below are therefore averages over the 50 randomly selected training and testing sets. Since the output of the datasets is either 0 or 1, this is a binary classification problem.

Let *TP*, *FP*, *TN* and *FN* denote the number of true positives, false positives, true negatives and false negatives, respectively. Then, the performance metrics that we use to evaluate the results are the Sensitivity (TP/(TP+FN)), the Specificity (TN/(TN+FP)) and the BalancedAccuracy, denoted for short BA ((Sensitivity+Specificity)/2).

## 3. Classification Results

In the 37 datasets corresponding to the 37 assays, the ratio between positive and negative compounds varies between 5% and 30% with a mean around 12%. This highlights the unbalanced property of the data in the favor of negative compounds. Here, we test the ML algorithms on these unbalanced data and after balancing using data augmentation.

### 3.1. Results on Unbalanced Datasets

**Figure 1:**
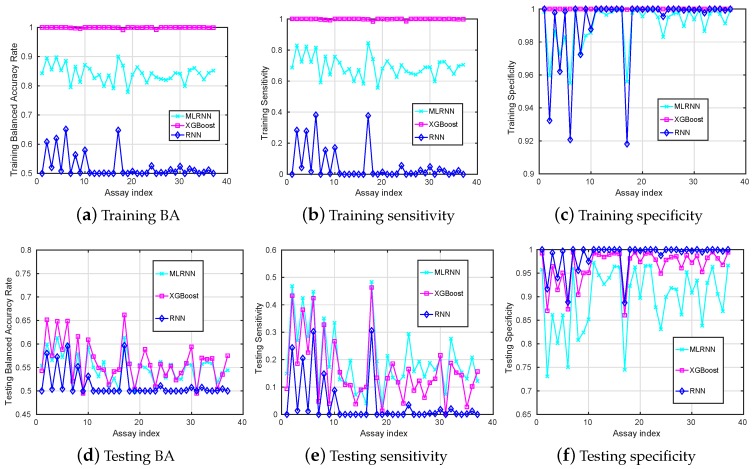
Training (**a**–**c**) and testing (**d**–**f**) mean-value results (*y*-axis) versus different assays (*x*-axis) when the MLRNN, XGBoost, RNN are used for classification.

The MLRNN, RNN and XGBoost algorithms are exploited to classify the 50×37 pairs of training and testing datasets and results are summarized into Figure 1. Since these are unbalanced datasets, the BA may be a better metric to demonstrate the classification accuracy. In addition, the situation of misclassifying positive as negative may be less desirable than that of misclassifying negative as positive. Therefore, the metric of Sensitivity is also important.

When looking at the BA obtained on the training data set in Figure 1a, we observe that the RNN method is not good at learning from these unbalanced datasets, while the MLRNN and XGBoost techniques learn much better.

Compared to the training accuracy, the performance on the testing dataset is more important since it demonstrates whether the model generalises accurately with regard to classifying previously unseen chemical compounds. The testing results are presented in Figure 1d–f. Here, we see that RNN performs the worst in identifying true positives (Sensitivity) and tends to classify most unseen chemical compounds as inactive, except for some assays. It can be explained by the overall number of inactive compounds much larger than the number of active compounds in the training dataset. The MLRNN and XGBoost perform a bit better in identifying the TPs, and the MLRNN performs the best. However, Sensitivity is still low and really depends on the assays and probably on the balance between active and inactive compounds in the corresponding datasets.

Among all assays, the highest testing BA achieved by these classification tools is 66.19% attained by the XGBoost for assay number 17, with the corresponding Sensitivity being 46.32%. Among all assays, the highest testing Sensitivity is 47.75% (MLRNN for assay 17) with a corresponding BA of 60.80%.

### 3.2. Results on Balanced Datasets

From the previous results, it appears that most of the classification techniques used are not good at learning unbalanced datasets. Therefore, we try balancing the 50×37 training datasets with data augmentation, while the corresponding testing datasets remain unchanged.

**Figure 2:**
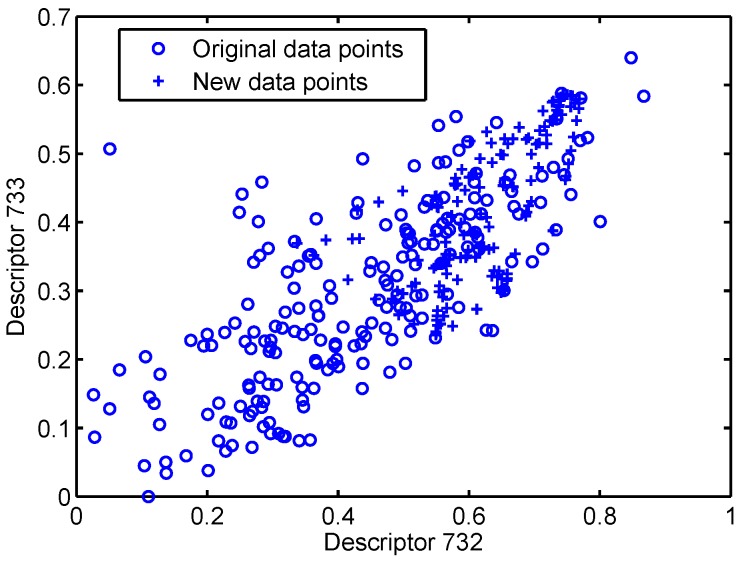
Two-descriptors plot of the training dataset after data augmentation.

**Figure 3:**
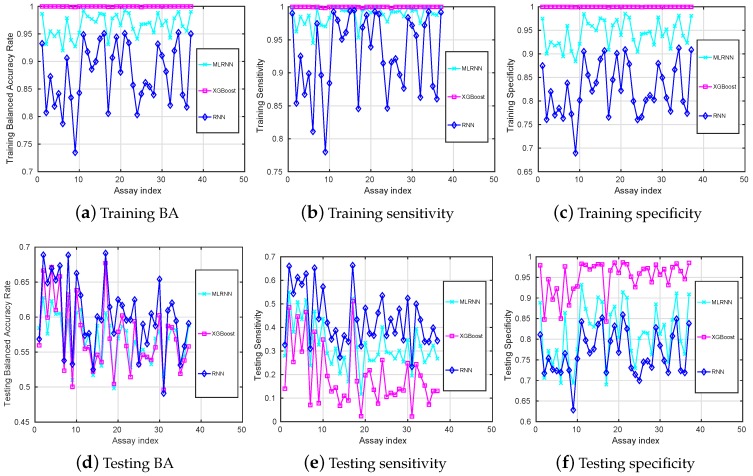
Training (**a**–**c**) and testing (**d**–**f**) mean-value results (*y*-axis) versus different assays (*x*-axis) on balanced datasets.

Here, the MLRNN, RNN and XGBoost are used to learn from the 50×37 datasets which are augmented for balanced training using the SMOTE method [54] as implemented in the Python toolbox *unbalanced_learn* [55]. Specifically, we plot two descriptors (Descriptors 732 and 733) of the training dataset after data augmentation in Figure 2. We can see that new samples are generated based on the original ones, and added to the dataset. Since the new points are correlated with the existing original points, this could be called “oversampling” (because of the correlation) or “augmentation” because the added points do not exist in the original dataset. The resulting Sensitivity, Specificity and BA are summarised in Figure 3.

Compared to the training balanced accuracies given in Figure 1a, Figure 3a shows that it is now evident that all the classification techniques we have discussed are capable of learning the training datasets after data augmentation. The training BA of the RNN method is still the lowest, but its testing BA is the highest for most of the assays.

Among all assays, the highest testing BA is 68.88% which is obtained with the RNN for the assay 17, with the corresponding testing Sensitivity being 66% and which is also the highest testing Sensitivity observed. Note that these values are higher than those reported in Figure 1.

**Figure 4:**
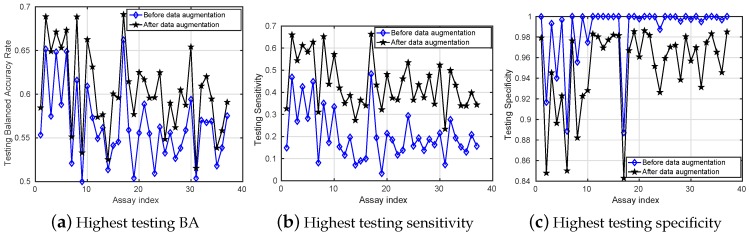
Comparison between the highest testing results (*y*-axis) versus different assay index (*x*-axis) on both unbalanced and balanced datasets. The interpretation of the results in this figure should be viewed as “heuristic” since a careful interpretation would require a detailed analysis of the statistical confidence intervals for each case.

Finally, for a better illustration, Figure 4 compares the highest testing results obtained among all classification tools for classifying the datasets before and after data augmentation. This figure highlights the clear improvement of Sensitivity for all assays, which also leads to a better BA for most of them. Not surprisingly, Specificity is decreased after data augmentation since the proportion of negatives in the balanced training sets is much lower compared to the original ones. Therefore, the models do not predict almost everything as negative as they did before data augmentation.

## 4. Conclusions and Perspectives

From the results presented here, we can draw several conclusions. First, the methods we have proposed can correctly predict bioactivity from the physico-chemical descriptors of compounds. However, some methods appear to be significantly better than others. In addition, the capacity to build good models seems to depend strongly on the assays themselves and their corresponding datasets. Moreover, we see that data augmentation techniques can play an important role in classification performance for the unbalanced datasets.

This work on ML applied to toxicology data raises further interesting issues. Since there is no absolute winner among the classification techniques that we have used, we may need to test other methods such as Support Vector Machines (SVM) [56] or Dense Random Neural Networks (DenseRNN) [57]. In addition, it would be interesting to apply the algorithms used on this small dataset to a larger one. We may also test other data augmentation techniques to seek the most appropriate ones [58]. Futhermore, in order to assess the prediction accuracy of bioactivity for a new compound, it is important to know if this compound has a chemical structure that is similar to the ones used in the training set. For this, we could use the “applicability domain” approach [59] as a tool to define the chemical space of a ML model.

If we refer to the long term objective of this work which is to link the molecular structure to in vivo toxicity, we could think about using the approach we have used as an intermediate step, and also train ML techniques to go from in vitro data to the prediction of in vivo effects. However, some preliminary tests that we have carried out (and not yet reported) reveal a poor correlation between in vitro and long term in vivo results. Therefore, it is necessary to find in vitro assays that are really informing about in vivo toxicity before considering them in future ML predictive models. In addition, we could consider combining the results obtained with several ML methods, similar to a Genetic Algorithm based combination [60,61], to enhance the prediction accuracy.

Finally, future work could also consider using more powerful G-Network models for learning, such as those [62] directly inspired from G-Networks with triggered customer movement [4], and models with strong inhibition such as “batch removal” [5].

## Abbreviations

The following abbreviations are used in this manuscript:

ML	Machine Learning
RNN	Random Neural Network
QSAR	Quantitative Structure–Activity Relationship
MLRNN	Multi Layer RNN
TP	True Positive
TN	True Negative
FP	False Positive
FN	False Negative
BA	Balanced Accuracy
